# A conserved two-component system senses intracellular iron levels and regulates redox balance in *Mycobacterium* spp.

**DOI:** 10.1128/spectrum.01106-24

**Published:** 2024-09-24

**Authors:** Rahul Yadav, Deepak Kumar Saini

**Affiliations:** 1 Department of Developmental Biology and Genetics, Indian Institute of Science, Bangalore, India; 2 Department of Bioengineering, Indian Institute of Science, Bangalore, India; University of Dundee, Dundee, United Kingdom

**Keywords:** oxidative stress, heme, iron metabolism, two-component regulatory systems, redox signaling

## Abstract

For bacteria, an intricate coordination between sensing and regulating iron levels and managing oxidative stress is required as their levels are tightly interlinked. While various oxidative stress and heme-based redox sensors have been reported for both pathogenic and non-pathogenic bacteria, the mechanisms governing the modulation of intracellular iron levels in response to changes in redox status remain unclear. In this study, a gene-inactivated strain of mycobacterial sensor kinase PdtaS showed dysregulated expression of genes associated with iron metabolism, including Fe-S clusters, NADH dehydrogenases, and iron uptake. The strain showed poor growth in nutrient-limiting conditions, a defect rescuable by heme but not by Fe^3+^ supplementation. This observation was associated with the PAS domain of the PdtaS sensor kinase. Biochemical and biophysical experiments established heme-binding to the PAS domain and its inhibitory effect on PdtaS auto-kinase activity, suggesting that the absence of heme induces activation of this sensor kinase. Interestingly, despite having an endogenous heme biosynthetic pathway or even external heme supplementation, the ∆*pdtaS* mutant exhibited persistent low intracellular iron levels concomitant with elevated oxidative stress. Antioxidant supplementation mitigated growth defects, emphasizing the link between oxidative stress, intracellular iron levels, and PdtaS activity. RNA-IP identified key targets associated with redox homeostasis and iron metabolism as targets of the PdtaR response regulator. The study proposes a novel role for the PdtaS-PdtaR TCS in sensing heme, regulation of intracellular iron levels, and redox balance.

**IMPORTANCE:**

The research article investigates the intricate interplay between bacteria’s ability to take and utilize iron without inducing excess iron’s toxic effects, including oxidative stress. The study shows that bacteria achieve this by sensing intracellular iron available as heme through a sensory protein PdtaS, which turns off when heme is in excess and prevents iron uptake and iron efflux. The process shields bacteria from generating Fe-dependent free radicals and allows it to maintain viability. The absence of sensor kinase abrogates all these processes, increasing bacteria susceptibility to ROS and thereby slowing growth. This feature of the sensor kinase PdtaS makes it an attractive co-therapeutic target for tuberculosis therapy, where its inhibition will prevent iron uptake, even in the presence of low iron, thereby halting bacterial proliferation.

## INTRODUCTION

Iron-bound protoporphyrin IX, commonly known as heme, is a pivotal form of iron in biological systems, acting as an essential cofactor for a myriad of enzymes involved in fundamental cellular processes. Heme is central to activities such as oxygen transport, electron transfer, and responses to oxidative stress (1, 2). Iron availability is a critical growth-limiting factor in bacteria, prompting the tight regulation of chelatable free iron pools (3, 4). Two distinct processes, heme-based and non-heme-based mechanisms, govern iron acquisition in bacteria, each essential for survival (4, 5). Moreover, salvaged host heme represents a vital iron source for several pathogenic bacteria (3, 4). Upon degradation facilitated by enzymes like heme oxygenase (HO), heme releases iron or heme can also be utilized directly for the biosynthesis of heme-containing proteins (6–8). In the case of *Mycobacterium tuberculosis*, an analogous enzyme called MhuD (Rv3592, Mycobacterial heme utilization Degrader) is responsible for this heme degradation. MhuD can bind two heme molecules in its inactive state, suggesting a potential role as a heme storage protein (9, 10).

While heme is essential for various cellular processes, its excess levels can lead to heme-mediated toxicity due to the generation of reactive oxygen species (ROS) (6). In addition to heme, NADH-dependent dehydrogenases also play an important role in maintaining intracellular oxidative stress by catalyzing redox reactions that convert NADH to NAD^+^ and generate superoxide radicals in the process (11). Interestingly, bacteria use iron-dependent regulatory systems to counteract oxidative stress, which includes expressing various antioxidant proteins and regulatory proteins, such as two-component signaling systems (TCSs). TCSs, composed of sensor histidine kinases and response regulators, enable bacteria to sense changes in the levels of various internal and external cues, such as heme, oxygen, redox, etc., and modulate gene expression in response to the changes, thereby mounting an adaptive response (12–14).

In pathogenic bacteria such as *M. tuberculosis*, two-component signaling systems (TCSs) play a crucial role in various stages of its lifecycle and pathogenesis, including host cell invasion, intracellular survival, phagolysosomal fusion, dormancy, and cell division (15). Given their absence in mammals and their significance in *Mtb* physiology, TCSs have been proposed as promising targets for anti-tubercular therapy (15, 16). The genome of *Mtb* encodes 12 paired TCSs and 4 orphan response regulators (RRs) that do not have known partner sensor kinases (SKs) (16). One of the functionally paired but genetically separate TCS which is well conserved in both pathogenic and non-pathogenic mycobacteria is PdtaS-PdtaR two-component system, composed of sensor kinase PdtaS (Rv3220c) and the response regulator PdtaR (Rv1626) (17). This system was previously shown to be involved in nutrition and c-di-GMP sensing through its GAF domain (18). This study examines the role of the PdtaS-PdtaR two-component system, conserved in all *Mycobacterium* spp., in heme sensing and regulating oxidative stress response. Notably, PdtaS, a cytosolic sensor kinase (17), is found to intricately participate in heme sensing by interacting with it through a PAS domain present in its sensing module, which, in turn, inhibits its autokinase activity. Through a comprehensive analysis, our investigations highlight the novel and previously unanticipated role played by PdtaS-PdtaR signaling system in regulating iron metabolism and redox regulation in *Mycobacterium* spp. using *M. smegmatis* as model.

## RESULTS

### PdtaS sensor kinase mutant strain growth defect is rescued by heme supplementation

To understand the nature of signal perception and transduction through the PdtaS-PdtaR two-component signaling system, this study was initiated by analyzing the available gene expression data of a PdtaS KO strain of *M. smegmatis* to identify potential molecular triggers initiating the signaling from PdtaS to PdtaR. The analysis revealed notable differential expression of genes associated with Fe-S clusters, NADH dehydrogenases, iron uptake, synthesis, and degradation, notably MmpLs, MBTs, and ferrochelatase (Fig. S1) (18). These observations strongly hinted toward a diminished intracellular iron level in the Δ*pdtaS* strain. Building upon prior evidence demonstrating growth defect in ∆*pdtaS* cells in minimal medium, a key observation emerged, wherein the growth impairment could be ascribed to intracellular iron scarcity. To address this, initial investigations were aimed to elucidate the outcome of supplementing the minimal medium with diverse iron sources, encompassing both cell-permeable hemin-based (hemin chloride, as the hemin donor) and non-hemin-based sources (ferric ammonium citrate as the Fe^3+^ donor and ferrous ammonium sulfate as the Fe^2+^ donor).

**Fig 1:**
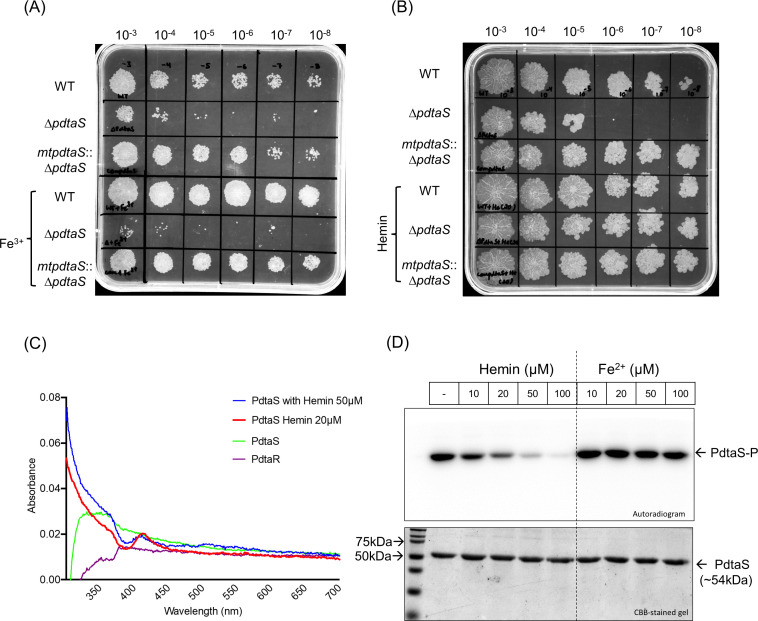
Effect of hemin and Fe^2+^ on PdtaS sensor kinase. (A, B) Growth analysis of 36-h cultures grown in minimal medium or minimal medium supplemented with (A) 20 µM ferric ammonium citrate and (B) hemin chloride. (C) UV-visible spectroscopy analysis of PdtaS in the presence or absence of hemin chloride. Hemin chloride alone was used as blank and representative spectra were plotted after subtracting the values of hemin absorbance. PdtaR was used as a negative control. (D) The effect of hemin and Fe^2+^ on PdtaS auto kinase activity was examined using an autophosphorylation assay. The data shown are representative of three independent experiments (*n* = 3).

The ∆*pdtaS* strain displayed a severe growth defect in the minimal growth medium, which could not be rescued by ferric ammonium citrate (Fig. 1A); however, when grown in the minimal medium with hemin chloride, the growth defect was lost (Fig. 1B). This observation underscores the ability of heme-based iron sources in facilitating the growth in the absence of the PdtaS histidine kinase. The rescue also pointed toward a possible regulation of PdtaS activity either by heme or by iron. PdtaS has been shown previously to interact with copper and c-di-GMP (18, 19). With this background, we probed if heme and Fe^2+^ can directly interact with PdtaS. For direct heme-binding analysis, UV-visible spectroscopy was utilized to validate the interaction of PdtaS with hemin. In the presence of hemin, PdtaS exhibited a distinctive peak at 412 nm, signifying its interaction with hemin, which was absent for both the PdtaS and PdtaR proteins (Fig. 1C). Furthermore, PdtaS displayed peaks in the 360–390 nm range in the presence of Fe^2+^, indicating an interaction with oxidized iron (Fig. S2).

With potential ligands identified, the next aim was to determine whether they can influence the activities viz. autophosphorylation of the PdtaS histidine kinase. To address this, PdtaS autophosphorylation assay was performed in the presence of hemin, or a Fe^2+^ donor. It was observed that while the ferrous donor did not affect the auto-kinase activity of PdtaS, hemin had a dose-dependent inhibitory effect (Fig. 1D). These key observations were also confirmed using purified *M. smegmatis* PdtaS protein (MSMEG_1918) to confirm that functional conservation among homologous proteins (Fig. S3). Our findings, thus, suggest that the absence of heme which leads to low intracellular iron activates the PdtaS sensor kinase leading to its autophosphorylation and the presence of free heme deactivates it and inhibits autophosphorylation, an observation also recorded for VgrRS TCS with ferrous/ferric iron (20).

**Fig 2:**
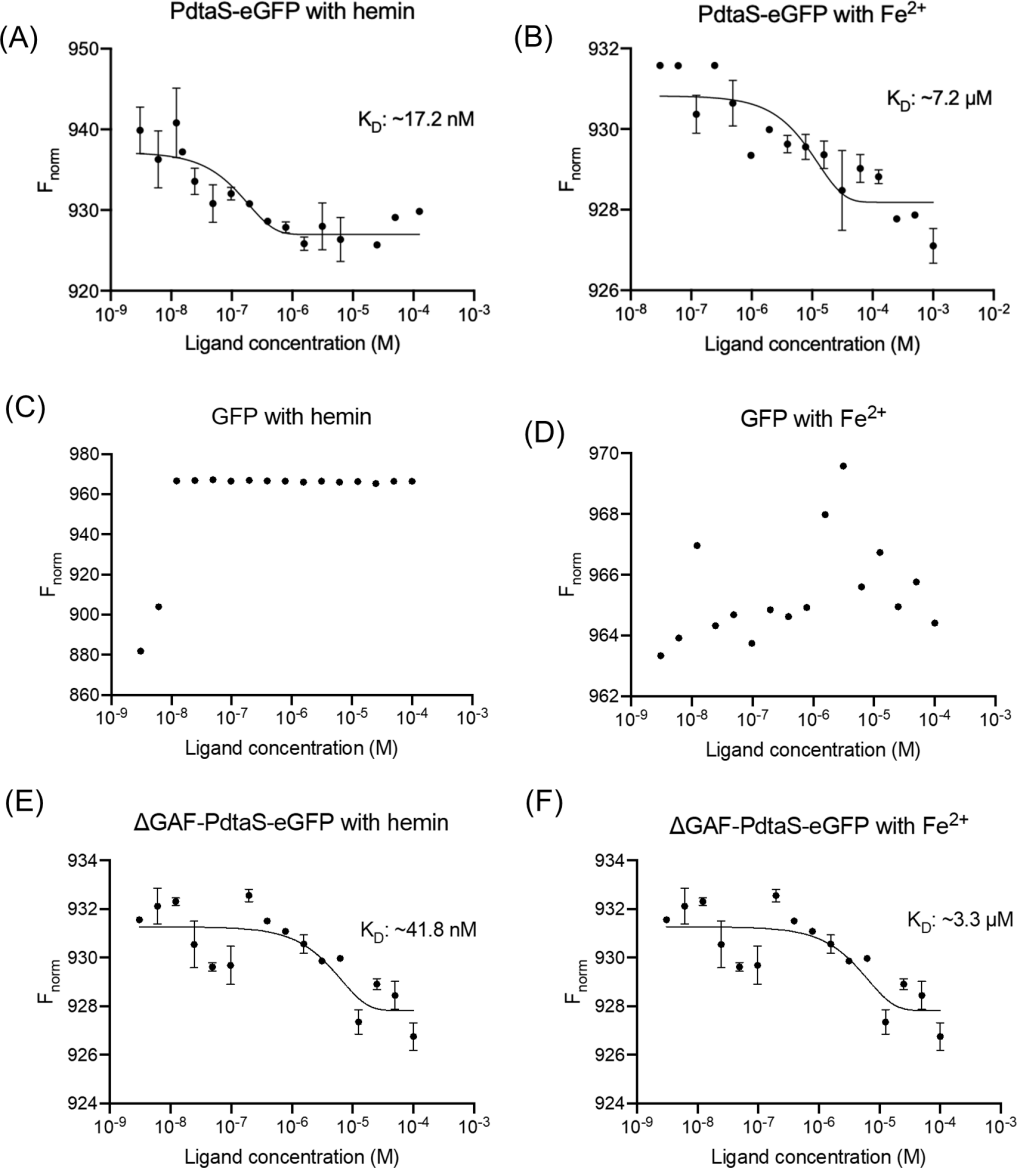
Interaction analysis of hemin and Fe^2+^ with PdtaS sensor kinase. Normalized fluorescence intensity obtained from microscale thermophoresis experiment of GFP-tagged PdtaS, and-∆GAF-PdtaS to record interaction with putative ligands. (**A**) PdtaS-eGFP interaction analysis with hemin (15.25 nM to 50  µM), (**B**) PdtaS-eGFP with Fe^2+^ (30 nM to 1 mM), (**C**) GFP with hemin (3 nM to 0.1 mM), (**D**) GFP with Fe^2+^ (3 nM to 0.1 mM), (**E**) ∆GAF-PdtaS-eGFP with hemin (3 nM to 0.1 mM), (**F**) ∆GAF-PdtaS-eGFP with Fe^2+^ (3 nM to 0.1 mM), Curves are best-fits, and symbols are mean  ±  SEM (*n*  ≥ 2 independent experiments).

To determine the interaction affinities of hemin and Fe^2+^ with PdtaS sensor kinase, microscale thermophoresis (MST) analysis was performed. Interestingly, these experiments revealed that both hemin (hemin chloride) (Fig. 2A) and Fe^2+^ (ferrous ammonium citrate) (Fig. 2B) interact with PdtaS-eGFP at binding affinities (*K*_D_) of approximately 17.2 nM and 7.2 µM, respectively. To confirm the specificity of hemin and Fe^2+^ interactions with the PdtaS protein, binding was tested with GFP alone as a control (Fig. 2C and D). Furthermore, given that PdtaS sensor kinase contains GAF, PAS, and conserved kinase domains, to identify the domain heme or Fe^2+^ interacts with, domain deletion variants of PdtaS protein were used. The ∆GAF-PdtaS-eGFP protein lacks the GAF domain, and ∆GP-PdtaS-eGFP lacks both the GAF and PAS domains. The analysis revealed that unlike ∆GP-PdtaS-eGFP (Fig. S4A and B), ∆GAF-PdtaS-eGFP interacts with both hemin (Fig. 2E) and Fe^2+^ (Fig. 2F) with an observed dissociation constants (*K*_D_) for hemin and Fe^2+^ as approximately 41.8 nM and 3.3 µM, respectively. These findings confirm that the hemin is an inhibitory ligand for the PAS domain of PdtaS sensor kinase.

Interestingly, while c-di-GMP is reported to be an activator for PdtaS sensor kinase (18), this study shows hemin as an inhibitor. To address this antagonistic effect, the impact of varying concentrations of hemin chloride, in the presence of c-di-GMP, was tested on PdtaS activity using an autophosphorylation assay. Surprisingly, the findings revealed that hemin exerted a dominant influence over c-di-GMP, resulting in a dose-dependent inhibition of PdtaS activity (Fig. S5). Interestingly, when a growth assay was conducted in a minimal medium supplemented with 100 µM hemin chloride, there was a complete absence of growth (Fig. S6) in all strains, mimicking the absence of PdtaS sensor kinase. This unique concentration-dependent role of hemin supported further interrogation on the impact and levels of iron in the knockout strain and how the presence of hemin makes PdtaS-PdtaR signalling redundant.

### Role of PdtaS sensor kinase in regulating intracellular iron levels

Based on the observations above, it was evident that there is dysregulation of intracellular iron levels in the ∆*pdtaS* strain, but it was not clear how PdtaS-PdtaR TCS system regulates iron homeostasis. To unravel this, we performed quantitative real-time RT-PCR (qRT-PCR) to assess the transcriptional changes in genes associated with iron metabolism in the mutant strain in the presence of various iron sources. The genes which were specifically examined included *bfrA*, a putative bacterioferritin involved in efficient iron utilization under low iron conditions; *bfrB*, a ferritin-like iron storage protein; *ideR*, an iron-dependent regulator responsible for intracellular iron homeostasis; *mhuD*, responsible for mycobacterial hemin utilization degradation; *hupB*, a potential heme importer; *mmpL3*,*L11*, which facilitate hemin import as well as *mmpS4*, *mmpL4*, *mmpS5*, and *mmpL5*, genes involved in siderophore export (5, 21).

**Fig 3:**
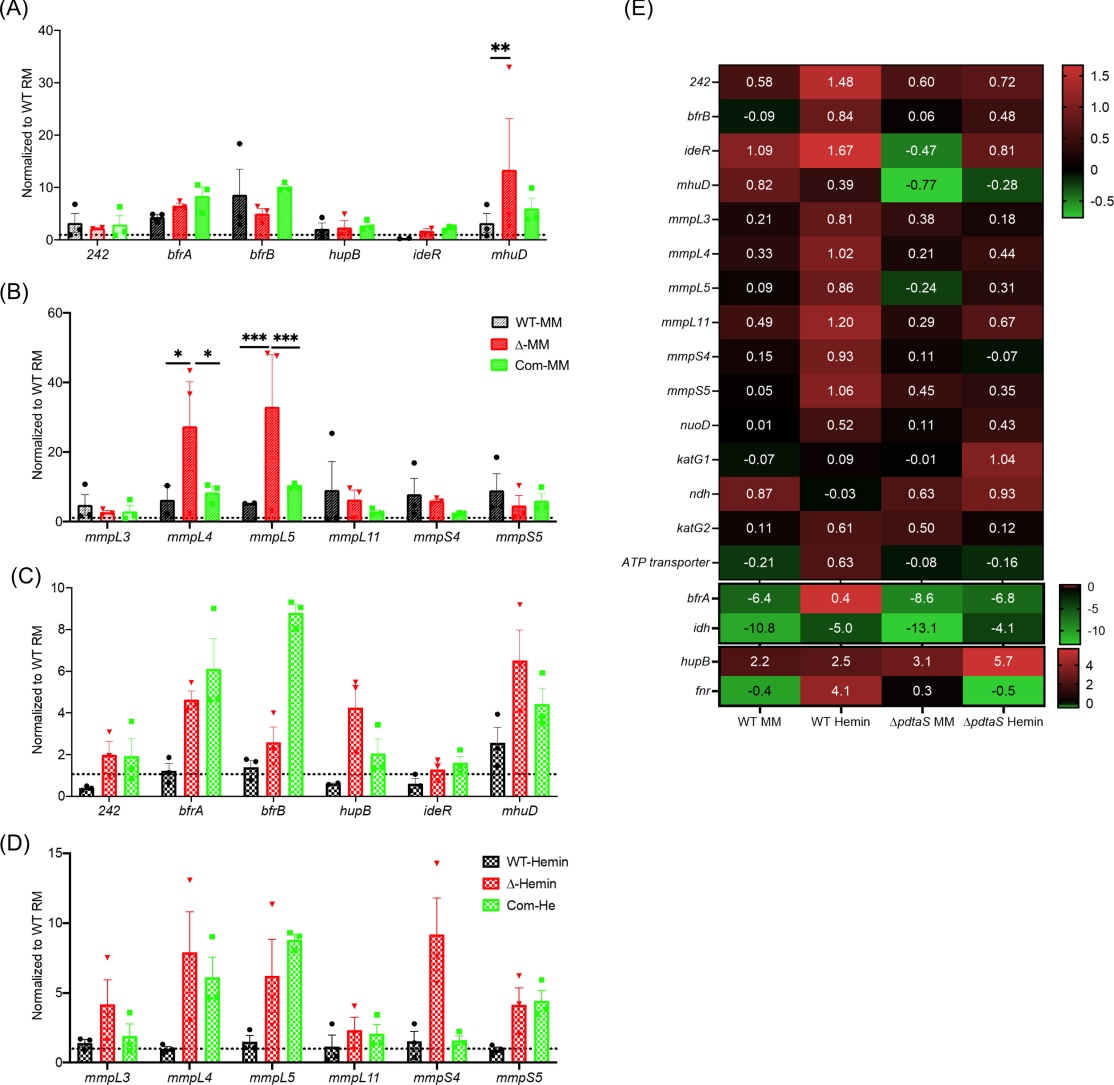
Expression analysis of genes involved in iron metabolism in various growth conditions. Expression analysis of iron acquisition genes in various strains as indicated. qRT-PCR was performed on RNA isolated from WT *M. smegmatis* and Δ*pdtaS* strains grown in rich medium, minimal medium, and minimal medium supplemented with hemin chloride. The black bars represent WT cells, the red bars represent ∆*pdtaS* strain, and the green bars represent ∆*pdtaS* strain complemented with *mtpdtaS*. (**A, B**) Change in expression in minimal media, and (**C, D**) expression changes in the presence of hemin. (**E**) Heat map generated using the changes in the expression levels of genes associated with iron acquisition, metabolism, and detoxification between WT and Δ*pdtaS* strain grown in rich medium and minimal medium. The expression levels of genes are normalized to WT-RM cells as 1. The fold change depicted is the mean of three independent experiments, with error bars representing the standard error of the mean. Statistical analysis was performed using ordinary two-way ANOVA (Dunnett’s multiple comparisons test). ****P < 0.0001; ***P < 0.001; **P < 0.01; and *P < .05 .

Toward these analyses, for baseline expression levels, the wild-type (WT) and ∆*pdtaS* strains were cultured in a nutrient-rich medium (7H9 with ADC). When ∆*pdtaS* strain was grown in a minimal medium, there was a significant upregulation of genes related to siderophore export *mmpL4* and *mmpL5*, and *mhuD* (Fig. 3A and B). This observation supported our low intracellular iron hypothesis as upregulation of *mmpL4*, and *mmpL5* is associated with low intracellular iron levels (21). The expression levels of *mmpL4* and *mmpL5* were significantly downregulated in hemin-supplemented ∆*pdtaS* cells compared to PdtaS knockout cells grown in the minimal medium alone (Fig. 3C and D), whereas expression levels of almost all genes in hemin-supplemented cells were comparable to WT cells grown in rich medium (Fig. 3C and D) indicating that the defect in ∆*pdtaS* strain is associated with low intracellular iron which was reversed by hemin supplementation. Intriguingly, the expression levels of iron acquisition genes in the supplemented medium still showed a disparity between WT and ∆*pdtaS* cells, emphasizing the role of PdtaS-PdtaR in the regulation of iron acquisition genes (Fig. 3C and D).

In a typical TCS, the target genes are regulated by the response regulator (RR) protein. Thus, to identify the potential targets of PdtaR, the gene expression data (above) were transformed into a heat map for visual discrimination. The underlying hypothesis posited that certain genes might exhibit expression alteration following hemin supplementation in WT cells. If the ∆*pdtaS* strain also shows similar changes, it will suggest that PdtaS is dispensable for the regulation of these genes, as evidenced by the growth rescue observed in ∆*pdtaS*. For instance, *ideR* follows this pattern. Conversely, some genes may deviate from this trend, and expression change in mutant will differ from WT cells, highlighting the significance of PdtaS-PdtaR in their regulation. An example of this is *mhuD*, thus representing a potential target of PdtaR. Overall, based on the heatmap, *mmpL3*, *mmpS3*, *ndh*, *katG2*, *ATP transporter*, *fnr,* and *mhuD* follow similar trend and can be putative targets of PdtaR (Fig. 3E).

### Low intracellular iron levels in ∆*pdtaS* strain

**Fig 4:**
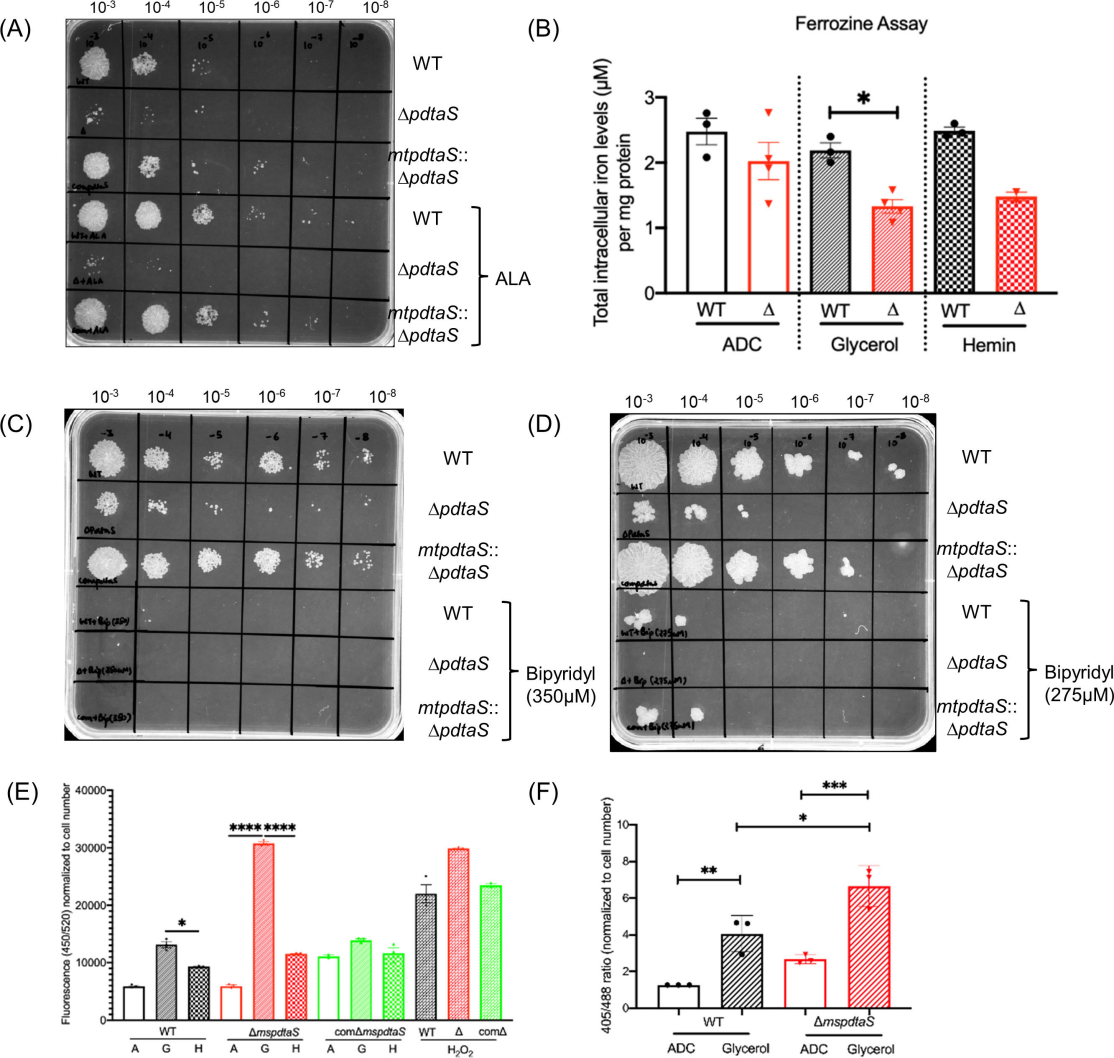
The ∆*pdtaS* strain has lower intracellular iron levels and higher intracellular oxidative stress. (**A**) Spot growth assay for various strains as mentioned, in minimal medium and minimal medium supplemented with δ-ALA (5 µg/mL). (**B**) Ferrozine assay for quantification of total intracellular iron content in WT and ∆*pdtaS* strains across various growth conditions, rich medium (7H9 with ADC), minimal medium (7H9 with 2% glycerol), and minimal medium supplemented with 20 µM hemin chloride. (**C**) Spot growth analysis of strains grown in minimal medium and minimal medium supplemented with 350 µM bipyridyl, or (**D**) 275 µM bipyridyl. (**E**) Measurement of intracellular oxidative stress using DCFDA in WT, ∆*pdtaS* (represented by ∆) and *pdtaS* complemented cells grown in 7H9 with ADC (**A**) or glycerol (**G**) or hemin (**H**). H_2_O_2_ was used as a positive control. (**F**) Measurement of intracellular oxidative stress using *mrx1*-roGFP2 sensor. The black bar represents WT cells, and the red bars represent ∆*pdtaS* cells. The filled pattern corresponds to the specific medium used, as indicated in the figure. Statistical analysis was performed using ordinary one-way ANOVA with Tukey’s multiple comparisons test. Error bars indicate the standard error of the mean (*n* ≥ 3). Significance is denoted as ****P < 0.0001; ***P < 0.001; **P < 0.01; and *P < 0.05. The data shown are representative of three independent experiments (*n* = 3).

To elucidate the underlying reason of the observed differences in gene expression between WT and mutant strain related to iron acquisition, an assessment of the intracellular iron levels was carried out. In comparison to the WT strain, Δ*pdtaS* displayed a notable reduction in intracellular iron levels in minimal medium, thus affirming that the deletion of the *pdtaS* gene, indeed, results in diminished intracellular iron content. However, a perplexing observation emerged when considering the anticipated increase in intracellular iron levels in minimal medium supplemented with hemin chloride. Surprisingly, under these conditions, Δ*pdtaS* still exhibited low intracellular iron content (Fig. 4B). Given that mycobacterium can synthesize heme through heme biosynthetic pathway and heme supplementation rescues the growth defects, an additional question arose about the functionality of heme biosynthetic pathway in ∆*pdtaS* cells. The examination of the hemin biosynthetic pathway led to the identification of aminolevulinic acid (ALA) as the primary committed intermediate within the common tetrapyrrole pathway (22). The hypothesis was formulated that if the growth deficiencies observed in Δ*pdtaS* were primarily attributed to a scarcity of iron, the supplementation of ALA would not restore growth as it also requires iron for final heme biosynthetic step. In agreement with this, when Δ*pdtaS* was grown in minimal medium supplemented with ALA, growth restoration was not observed (Fig. 4A). This observation suggested that Δ*pdtaS* cells did not need additional iron, which consequently led to suppression of the hemin biosynthetic pathway, and inducing growth defect.

To provide additional evidence for lower intracellular iron levels in the ∆*pdtaS* strain, growth analyses were performed in the presence of varying concentrations of 2–2′ bipyridyl, an iron chelator, in minimal medium. It was observed that concentrations exceeding 350 µM was lethal to both the WT and ∆*pdtaS* strains, signifying the cytotoxic nature of the iron chelator at elevated concentrations and underscoring the essentiality of iron for cell growth (Fig. 4C). However, at a concentration of 275 µM, the WT strain displayed diminished growth, unlike the ∆*pdtaS* mutant, thereby confirming lower intracellular iron levels in the absence of PdtaS (Fig. 4D).

### PdtaS deletion enhances intracellular oxidative stress

Based on the current findings, an obvious question emerges about factors which contribute to the low iron content observed in the ∆*pdtaS* strain despite its correlation with low growth and fitness. Iron plays a pivotal role in modulating oxidative stress due to its ability to catalyze the production of hydroxyl radicals (OH•) from hydrogen peroxide (H_2_O_2_) through the Fenton reaction (23, 24). Given that we observed reduced intracellular iron levels in the absence of PdtaS, it was hypothesized that the ∆*pdtaS* mutant might show concomitant alterations in the redox balance. To investigate this, a DCFDA assay was carried out to measure the intracellular oxidative stress. It was observed that the ∆*pdtaS* mutant exhibited higher levels of oxidative stress in comparison to the WT and *pdtaS* complemented cells in minimal medium (Fig. 4E). To further validate these findings, a well-established mycobacterial redox sensor mrx1_roGFP2 (25) was used, and the results confirmed that both the WT and ∆*pdtaS* strains exhibit heightened oxidative stress in minimal medium (Fig. 4F).

**Fig 5:**
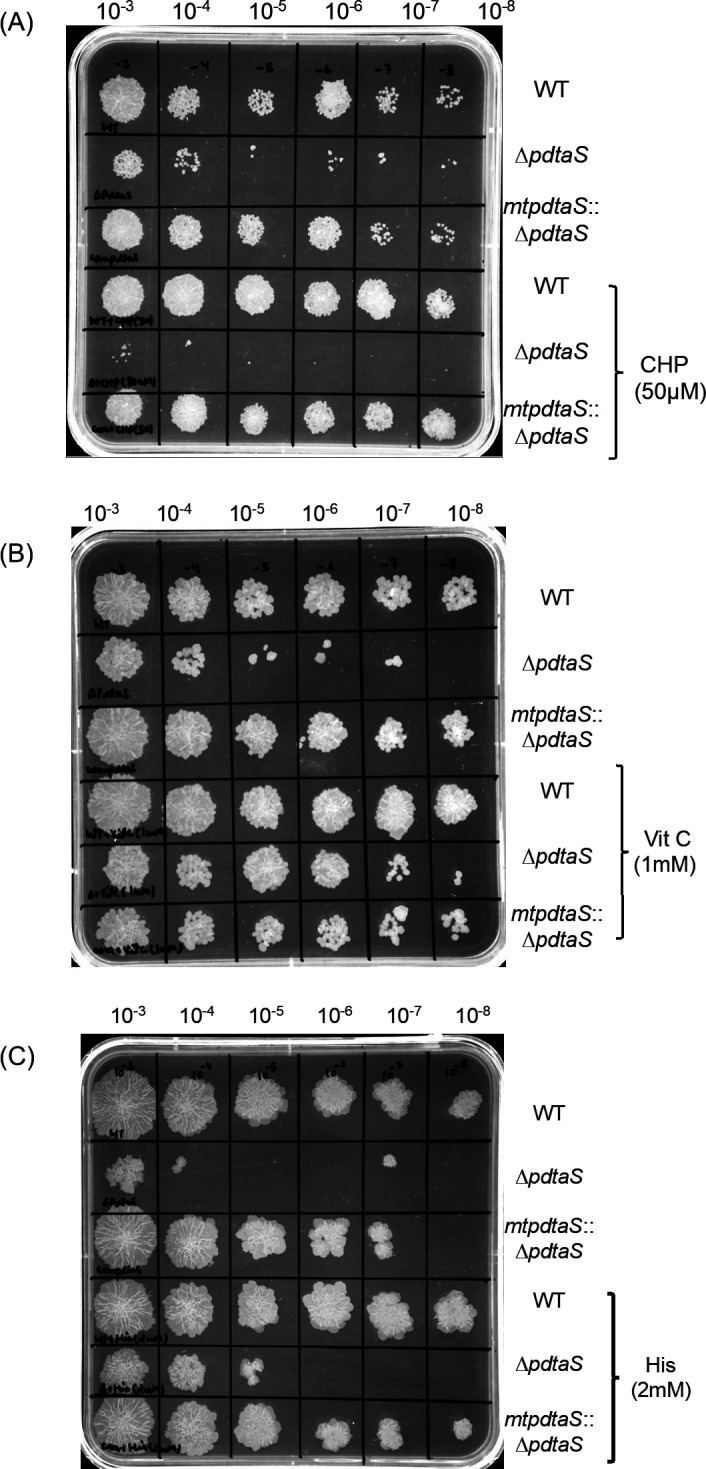
Effect of CHP, Vitamin C, and histidine supplementation on the growth of Δ*pdtaS* strain. Spot growth analysis of cultures grown in minimal medium and minimal medium supplemented with (**A**) CHP (50 µM), (**B**) 1 mM Vitamin C, and (**C**) 2 mM Histidine for various strains as indicated. The data presented are representative of three independent experiments (*n* = 3).

Given the elevated oxidative stress observed in the ∆*pdtaS* strain under minimal medium conditions, it was next investigated if this mutant is more susceptible to oxidative stress. To investigate this, the cells were subjected to various concentrations of an oxidative stress inducer, cumene hydroperoxide (CHP) in minimal medium. A severe impairment in the growth of the ∆*pdtaS* mutant, particularly when exposed to concentrations of ≥50 µM CHP, was recorded (Fig. 5A). Furthermore, at 100 µM CHP, growth was completely inhibited (Fig. S7) confirming the cytotoxic effect of CHP on all the tested strains.

To further validate that the observation that the growth impairment observed in the ∆*pdtaS* mutant is a direct consequence of elevated oxidative stress, growth analysis was performed in the presence of various antioxidant molecules. For this, the minimal medium was supplemented with 1 mM vitamin C (26), and as anticipated, its presence completely restored the growth of ∆*pdtaS* mutant strain (Fig. 5B). To reinforce these findings, another antioxidant, histidine was also tested at various concentrations, and partial recovery of the growth defect was recorded (Fig. 5C).

### *mhuD* and *nuoD* are phosphorylation-dependent regulatory gene targets for PdtaR response regulator

Classical signaling response in TCS signaling pathways involve modulation of activities of response regulator protein (here PdtaR) through a phosphorylation process triggered by the sensor kinase protein (here PdtaS) in response to the stimulus. Given that the phosphorylation of sensor kinase PdtaS is inhibited by heme, hence it is anticipated that the presence or absence of heme will modulate the activities of the partner response regulator protein PdtaR through a phosphorylation-dependent process.

Previously, it has been established that the PdtaR protein contains an RNA-binding ANTAR domain, which binds to RNA and serves as an anti-termination factor (27). ANTAR domain is also known to be preferentially linked to genes that affect lipid metabolism and the maintenance of redox homeostasis, such as dehydrogenases, oxidoreductases, and *ndh*, which encodes a non-proton pumping NADH dehydrogenase (27). Building upon these foundational insights, a substantial convergence in the regulation of redox processes, iron metabolism, and lipid-related cellular pathways mediated by the ANTAR domain of PdtaR is predicted (28).

**Fig 6:**
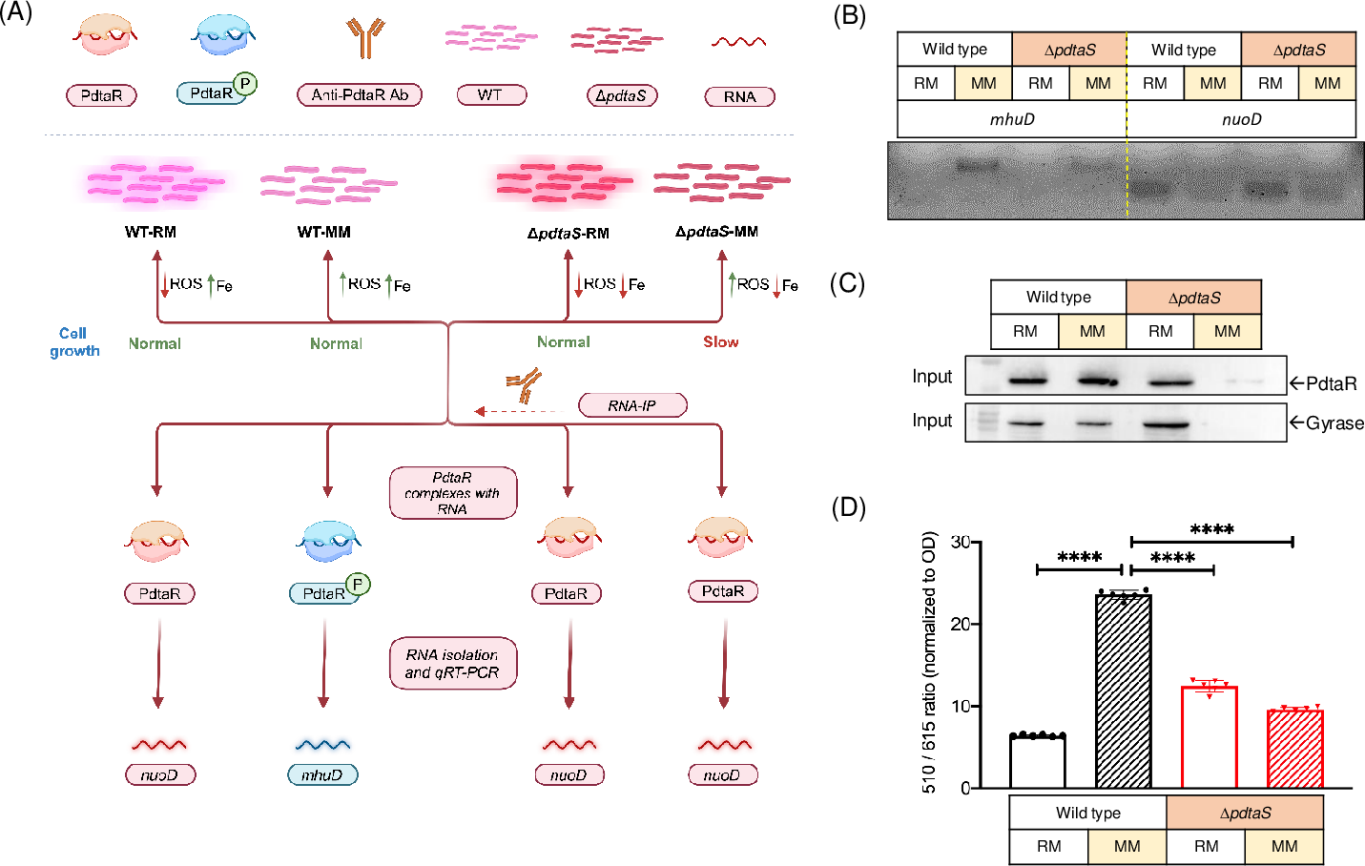
*mhuD* and *nuoD* as possible targets of PdtaR. (**A**) Schematic of RNA-IP and RNA targets identification procedure. The illustration delineates the key steps involved in identifying the targets of the PdtaR response regulator. Both WT and ∆*pdtaS* cells were cultured in rich and minimal media, resulting in four distinct combinations of intracellular oxidative stress and iron levels. WT-RM cells exhibited low levels of intracellular oxidative stress and high intracellular iron levels. Conversely, in minimal medium, WT cells displayed elevated intracellular oxidative stress compared to those in rich medium, alongside high iron concentrations. ∆*pdtaS* cells exhibited low intracellular iron levels under both conditions, with the highest oxidative stress observed in minimal medium. Anti-PdtaR antibodies were utilized for RNA immunoprecipitation (RNA-IP), followed by RNA isolation and quantitative real-time polymerase chain reaction (qRT-PCR). *nuoD* was identified as a target of PdtaR in its unphosphorylated state, while *mhuD* was identified as a target in its phosphorylated state. (**B**) Agarose gel electrophoresis demonstrating amplification of *mhuD* and *nuoD* transcripts across various experimental conditions after RNA-IP using anti-PdtaR antibodies. (**C**) Western blot analysis of input PdtaR protein levels in various lysates. PdtaR and Gyrase served as internal controls. Anti-PdtaR and Anti-Gyrase antibodies were used for detection. RM represents rich medium (7H9 with ADC) and MM stands for minimal medium (7H9 with 2% glycerol). (**D**) Measurement of intracellular NADH/NAD^+^ using peredox-mcherry sensor in pMV261 overexpression vector. The black bar represents WT cells, and the red bars represent ∆*pdtaS* cells. The filled pattern corresponds to the specific medium used, as indicated in the figure. Statistical analysis was performed using ordinary two-way ANOVA (Dunnett’s multiple comparisons test). ****P < 0.0001; ***P < 0.001; **P < 0.01; and *P < 0.05. The data presented are representative of three independent experiments (*n* = 3).

To validate this and identify potential targets of the PdtaR protein which links our observation of redox and iron homeostasis modulation, RNA immunoprecipitation experiments in conjunction with qRT-PCR were performed using anti-PdtaR antibodies and RNA from both the WT and ∆*pdtaS* cells grown in rich and minimal media. We aimed to identify putative genes which showed interaction with PdtaR in WT cells in both rich and minimal media growth conditions (model described in Fig. 6A). Through this approach, we successfully identified two specific target genes: *mhuD* in minimal medium (Fig. 6B; Fig. S8A) and *nuoD* in rich medium (Fig. 6B; Table 1).

**TABLE 1:** Role of PdtaS-PdtaR TCS in iron homeostasis and maintenance of intracellular oxidative stress^*a*^

Strain	Wild type	Wild type	∆*pdtaS*	∆*pdtaS*
Growth condition	Rich media	Minimal media	Rich media	Minimal media
ROS levels	Low	High	Low	High
Fe levels	High	High	Low	Low
Growth phenotype	Normal	Normal	Normal	Impaired
PdtaR status	Unphosphorylated	Phosphorylated	Unphosphorylated	Unphosphorylated
RNA target for PdtaR	*nuoD*	*mhuD*	*nuoD*	*nuoD*
Role of target	Regulates NADH/NAD^+^ ratio	Release of heme	Regulates NADH/NAD^+^ ratio	Regulates NADH/NAD^+^ ratio

^*a*^ The table summarises PdtaS-PdtaR TCS’s role in maintaining intracellular oxidative stress and iron levels, in the presence and absence of PdtaS sensor kinase.

To ensure that the protein levels of PdtaR used for IP were equal across the various conditions, western blotting was performed using anti-PdtaR, and anti-gyrase antibodies. The results indicated comparable PdtaR levels in each condition, with the exception of ∆*pdtaS* cells in a minimal medium (Fig. 6A and C), which was not completely unanticipated, as ∆*pdtaS* cells exhibit growth defects in a minimal medium, resulting in lower cell population. To address this issue, equal amounts of immunoprecipitated RNA were used for cDNA synthesis, and the qRT-PCR experiments were performed accordingly. Based on the RNA-IP data, it could be inferred that *mhuD* interacts with PdtaR in its phosphorylated state, while *nuoD* interacts with PdtaR in its unphosphorylated state (Table 1). Here *sigA*, *mtrA,* and *tcrX* were used as controls, and their transcripts were not enriched in any of the conditions tested (29) (Fig. S8B).

To further examine the impact of high oxidative stress in the PdtaS mutant strain, we examined the relative levels of nicotinamide adenine dinucleotide (NADH, reduced form; NAD^+^, oxidized form), a crucial cofactor that is involved in electron transfer in various metabolic processes, and plays a pivotal role in redox reactions. The NADH-dependent dehydrogenases (*ndh*) catalyze redox reactions leading to the conversion of NADH to NAD^+^. Building on these insights, we utilized a previously established peredox-mcherry sensor, previously shown to work in both slow- and fast-growing mycobacteria, to assess the cellular NADH: NAD^+^ ratio (11). The findings revealed that WT cells in rich medium and ∆*pdtaS* cells in both rich and minimal media exhibited a lower NADH: NAD^+^ ratio compared to WT cells grown in minimal medium (Fig. 6D), which indicated reduced levels of NADH dehydrogenases in the mutant strain.

**Fig 7:**
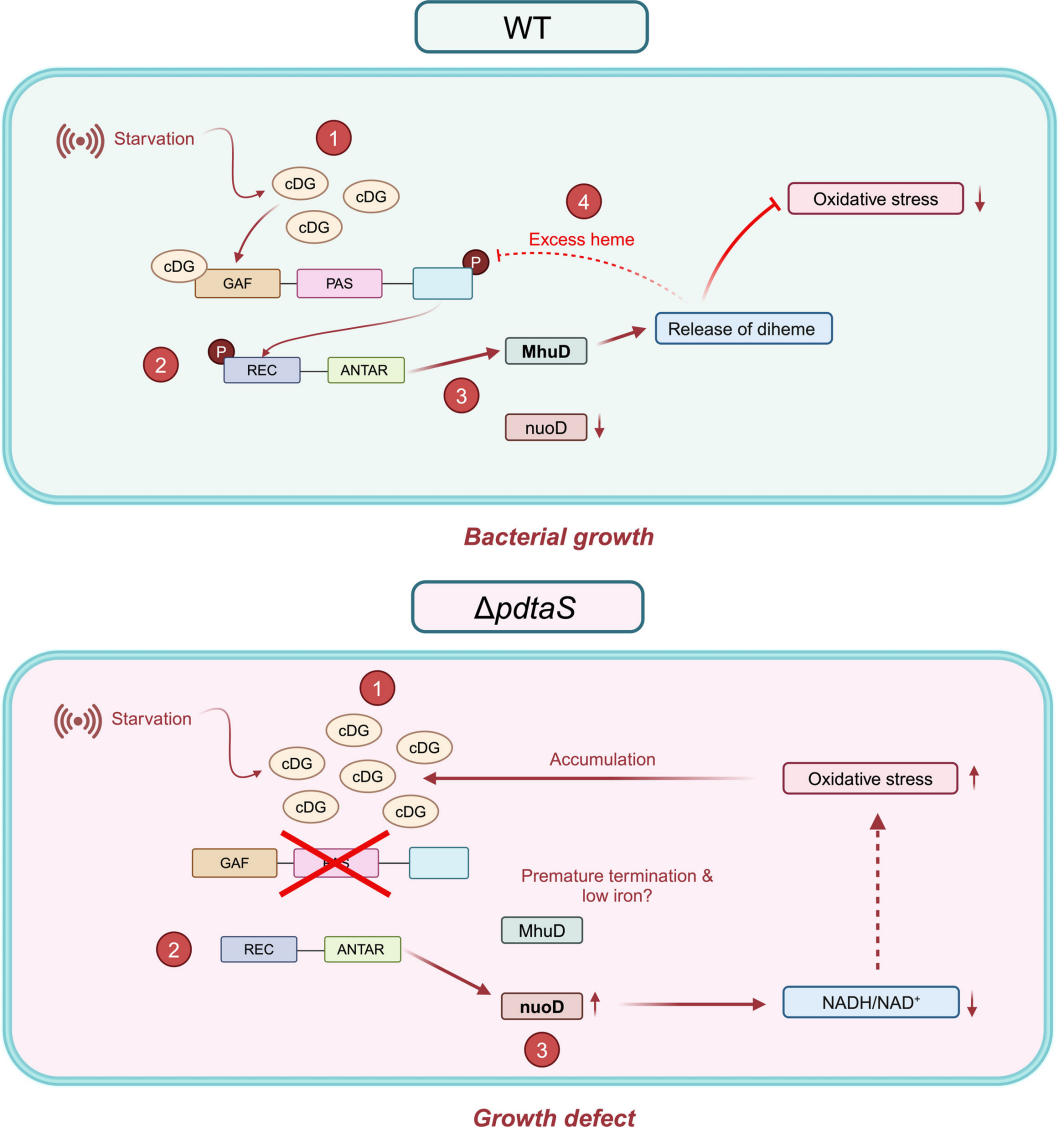
Proposed mechanism of PdtaS-PdtaR signaling in *Mycobacterium* in minimal medium. Step 1, in a minimal medium, c-di-GMP levels increase, which interacts with the GAF domain of PdtaS, leading to an increase in PdtaS phosphorylation. Step 2, phosphorylated PdtaS transfers the phosphoryl group to PdtaR. Step 3, phosphorylated PdtaR interacts with *mhuD*, whose gene products facilitate the release of diheme. Released diheme activates the expression of antioxidant genes, reducing oxidative stress. Step 4, in the presence of excess heme, PdtaS is inhibited through the interaction of heme with the PAS domain. In ∆*pdtaS* strain, the absence of PdtaS results in unphosphorylated PdtaR which can interact with NADH-dependent quinone dehydrogenases (*nuoD*), leading to the conversion of NADH to NAD^+^. This induces oxidative stress which, in turn, leads to c-di-GMP accumulation, and high intracellular oxidative stress leads to the growth defects observed in ∆*pdtaS* strain.

Overall, our findings provide valuable insights into the role of the PdtaS-PdtaR two-component system (TCS) in regulating redox and iron homeostasis. Our RNA-IP experiments indicate that PdtaR interacts with different targets based on its phosphorylation status (driven by the presence or absence of PdtaS protein), wherein *mhuD* interacts with PdtaR in its phosphorylated state and *nuoD* interacts in the unphosphorylated state. The model described in Fig. 7 details these processes wherein roles for c-di-GMP, iron, oxidative stress as a sensory modulator; PAS-GAF domains as sensory domains, and ANTAR-mediated regulation of *mhuD* and *nuoD* is described.

## DISCUSSION

The fitness of bacterial cells critically relies on their ability to perceive and adapt to alterations in the physical and chemical composition of their surroundings. To achieve this, bacteria deploy sensory and signal transduction proteins, such as two-component signaling proteins, which contain diverse input domains in the SK through which signals or stimuli are detected. Similarly, the response regulator protein has diverse output domains through which they link external stimuli to target genes (12).

The findings presented in this study shed light on the intricate interplay between oxidative stress, heme, and c-di-GMP sensing by PdtaS-PdtaR TCS in *Mycobacterium* spp. Central to this study is the observation that the absence of PdtaS results in lower intracellular iron levels accompanied by heightened oxidative stress and impaired growth, highlighting the crucial role of the PdtaS-PdtaR signaling pathway in cellular iron and redox homeostasis. One of the key findings of this study is the link between heme and PdtaS activity. Heme plays a pivotal role in the physiology of mycobacteria and serves as a key oxygen sensor in mycobacteria, influencing the activity of proteins like DevS and DosT. These sensor kinases are highly homologous and have identical architecture, with two N-terminal GAF domains, that has been shown to recognize environmental changes in the levels of diatomic gases such as O_2_, NO, or CO using heme as a cofactor (30, 31). In this study, we found that PdtaS directly interacts with heme which inhibits its kinase activity unlike DevS and DosT. The growth defects in ∆*pdtaS* cells in minimal medium and the changes in expression of iron metabolism genes were compensated by the presence of heme-based iron source, which rescued growth defect. This observation was biochemically backed by PdtaS binding with heme and its inhibitory effect on PdtaS autophosphorylation. This dual role of heme in facilitating bacterial growth and inhibiting PdtaS activity indicated that heme has a multi-pronged role in *Mycobacterium* (30, 31).

Heme as a prosthetic group is best known for roles in oxygen transport, oxidative catalysis, and respiratory electron transport. It has been shown that at low heme concentrations, heme oxygenase (MhuD in *M. smegmatis*) activity becomes more predominant. The by-products of heme degradation act as antioxidants, providing protection against oxidative stress (32), which supports the finding that ∆*mspdtaS* cells show rescue of growth defects and decrease in oxidative stress after 20 µM hemin chloride supplementation. Conversely, high concentrations of heme can increase iron release during heme breakdown. Heme homeostasis is crucial as free heme at >1 µM is cytotoxic, mainly by producing reactive oxygen species (6), which was observed in our study where *M. smegmatis* showed no growth when supplemented with 100 µM hemin chloride.

Given that the effects of changes in phosphorylation of sensor kinase are typically reflected as a change in the levels of target genes regulated by response regulators, the presence of heme, which ultimately inhibits the PdtaS, physiologically generates a PdtaS knockout strain but without growth defect, thereby allowing distinction of targets dependent on heme and those dependent on PdtaS-PdtaR system. A simple gene expression comparison of mutant stain in the presence or absence of heme, thus, revealed genes that depend on PdtaS for expression from those dependent on heme alone. This revealed dysregulated expression for genes related to siderophore export, namely, *mmpL4* and *mmpL5* and *mhuD* in the ∆*pdtaS* strain. MhuD modulates heme levels in *M. tuberculosis* to maintain sufficient levels of hemoproteins, while mitigating heme-induced toxicity (9, 33). These reports and our data supported the observation that ∆*pdtaS* cells have low intracellular iron levels because of the upregulation of *mmpL4* and *mmpL5*, which is necessary in the mutant stain to keep oxidative stress in check. This was a crucial observation as we recorded that despite the lower intracellular iron levels in ∆*pdtaS*, the mutant strain showed heightened oxidative stress, reflected by increased sensitivity to cumene hydroperoxide; growth rescue in the presence of antioxidants, such as Vitamin C (26), and alteration in NADH/ NAD^+^ ratios. The gene targets that lead to high oxidative stress and low iron were differentially regulated by response regulator PdtaR, through a phosphorylation dependent interaction. The unique aspect was driven by the regulation of different genes by PdtaR in the presence and absence of PdtaS.

The response regulator PdtaR contains a receiver and an ANTAR domain. In bacteria, the binding of ANTAR protein to RNA stabilizes a transcriptional antiterminator, thereby allowing the uninterrupted continuation of transcripts that would otherwise be prone to termination (27). It is noteworthy that some primary targets of ANTAR domain-containing proteins encompass dehydrogenases, putative luciferase-like oxidoreductases, and *ndh*, which encodes a non-proton pumping NADH dehydrogenase. It has been established that the presence of an ANTAR domain in proteins is preferentially linked to functions involving lipid metabolism and the maintenance of redox homeostasis (18, 28) This suggested that proteins harboring ANTAR domains can directly modulate cellular redox processes, which posits a substantial convergence in the regulation of redox processes, iron metabolism, and lipid-related cellular pathways mediated by ANTAR domains across various mycobacterial species. To prove these aspects, the targets of PdtaR response regulator which communicates with PdtaS sensor kinase were identified using RNA-IP. RNA-IP experiments revealed specific genes interacting with phosphorylated or unphosphorylated PdtaR, such as *mhuD* and *nuoD*, respectively.

In conclusion, this study advances our understanding of the PdtaS-PdtaR signaling pathway in *Mycobacterium* and how it regulates cellular iron homeostasis and oxidative stress response. The findings highlight the intricate regulatory mechanisms governing heme-mediated signaling, c-di-GMP modulation, and the interplay between oxidative stress and iron homeostasis.

### Limitations of the study

The study focuses on the model organism *M. smegmatis*. The findings may not fully translate to pathogenic mycobacteria like *M. tuberculosis* without additional validation. Environmental and physiological conditions in the study may not entirely reflect those encountered by mycobacteria in their natural habitats or host environments.

### Strength of the study

The study elucidates the critical role of the PdtaS-PdtaR signaling pathway in regulating cellular iron homeostasis and oxidative stress in *Mycobacterium* spp. It identifies heme as a dual regulator, influencing both bacterial growth and kinase activity of PdtaS, adding depth to the understanding of heme’s multifaceted role in mycobacterial physiology. This study also identifies ligands and targets of PdtaS-PdtaR TCS, advancing the knowledge of redox and iron metabolism regulation.

## MATERIALS AND METHODS

### Growth analysis for various *M. smegmatis* strains

Primary cultures of *M. smegmatis* strains were grown in TSB (unless otherwise specified) for 24 h or until the OD reached 0.8–1.0. For starvation experiments, primary cultures were washed thrice with 7H9 alone (no carbon source, no supplements) followed by resuspension in 7H9 alone for 8 h, following which they were sub-cultured at a starting OD of 0.005 in the medium used for the growth curve. To check the OD of cultures at specified time-points, 200 µL of cultures was pipetted into the wells of flat, transparent clear bottom 96-well plates (Corning, USA) in triplicate and the absorbance of the wells were measured at 600 nm using the bottom reading mode of the Infinite M1000 PRO multimode fluorescence plate reader (Tecan, Austria).

### Dilution spot assay for growth assessment

A single time point assay for the growth of *M. smegmatis* was standardized using spot dilutions on tryptone soy agar (TSA) plates. Briefly, primary cultures of *M. smegmatis* strains were inoculated in tubes containing the appropriate medium at an OD of 0.005. Cultures were allowed to grow for 36 h (mid-log phase for WT cells) following which serial 10-fold dilutions of cultures were made in 7H9 base medium. Five microliters of each dilution was spotted carefully on the surface of TSA plates without any antibiotics. Spots were allowed to dry (no visible moisture on the agar surface) following which they were incubated at 37°C for 2–3 days to allow for the growth of single colonies. Plates were imaged using Chemi-Doc MP (Bio-Rad, USA).

### Protein expression and purification

The recombinant proteins were expressed and purified as described in supplementary methods (17).

### Phosphorylation assays

Autophosphorylation and phosphotransfer assays were performed as described in supplementary methods (34, 35).

### Determination of hemin and Fe^2+^ binding affinity to sensor kinase PdtaS and its domain mutants by MST analysis

MST measurement to determine the affinity between the hemin and Fe^2+^ with the sensor kinase PdtaS and its domain mutants proteins was performed as described in supplementary methods. (18, 19, 36)

### Gene expression analysis

Analysis of changes in the expression levels of the genes was done from various strains by quantitative RT-PCR analysis as described in supplementary methods (18). RNA was isolated from 36 h grown cultures of WT *M. smegmatis*, and ∆*pdtaS* cells in rich medium, minimal medium, and hemin supplemented minimal medium. The primers used for qRT-PCR are listed in Table S1.

### Western blot analysis

For western blot analysis, 50–100 μg of total protein was resolved on 10% SDS-PAGE gel and transferred to PVDF membrane (GE Healthcare, USA) using a semi-dry transfer unit (Bio-Rad Inc, USA) at 20V for 12′. The membrane containing transferred protein was blocked with TBST buffer containing 5% BSA protein (Amresco, USA) for 1 h. The membrane was then incubated overnight with anti-PdtaR primary antibody diluted in TBST at 4°C on a slow rocker. Membranes were washed thrice for 10′ in TBST and incubated with appropriate HRP conjugated secondary antibodies (Jackson Laboratories Inc., USA) diluted as per manufacturer’s recommendations at room temperature for 1 h. Membranes were again washed thrice with TBST and subjected to chemiluminescent detection using ECL substrate (Western Lightning Plus, PerkinElmer, USA). The developed blots were imaged and analyzed using ChemiDoc MP Imaging system (Bio-Rad Inc., USA) at multiple cumulative exposure settings.

### RNA-IP

Protein A Sepharose 4 Fast Flow beads were equilibrated using 1 mL of IP buffer. This step was repeated thrice. The beads were blocked O/N at 4°C using 1% BSA prepared in IP buffer. It was followed by a wash with IP buffer, and then 10 µL (1–5 µg) of anti-PdtaR antibody was added. The total volume was always kept at 1 mL by making up the volume using IP buffer. This was kept for mixing for 4 h at 4°C. Beads containing anti-PdtaR antibodies were washed thrice, followed by the addition of cell lysate (~1 mg/mL), and kept for binding for 4 h at 4°C. After the final wash, the protein-bound beads were used for RNA isolation. For RNA isolation and qRT-PCR, the same protocol was used, as described above.

### Measurement of oxidative stress and NADH/NAD^+^ ratio using *mrx1*-roGFP2 and peredox-mcherry sensor

Primary cultures of WT and ∆*pdtaS M. smegmatis* strains having *mrx1*-roGFP2 (a kind gift of Dr Amit Singh, India) and peredox-mcherry (a kind gift of Dr Ashwani Kumar, India) sensors in pMV261 overexpression plasmid were inoculated in tubes containing the appropriate medium and antibiotics at an OD_600_ of 0.005. Cultures were allowed to grow for 36 h. After 36 h, cells pelleted and washed thrice with PBS/7H9. For NADH/NAD^+^ ratio, fluorescence readings were taken as 510/615 ratio (Ex/Em: 400 nm/510 nm and Ex/Em: 587 nm/615 nm). For oxidative stress, the ratio was 405 nm/488 nm (Ex/Em: 405 nm/510 nm and Ex/Em: 488 nm/510 nm).

### DCFDA (2′,7′-dichlorofluoresceinh) assay for intracellular ROS detection

Primary cultures of *M. smegmatis* strains were inoculated in tubes containing the appropriate medium at an OD of 0.005. Cultures were allowed to grow for 36 h. After 36 h, cells were pelleted and washed thrice with PBS/7H9. Cells were incubated in the dark at 37°C for 45 min with 10 µM DCFDA (Sigma Aldrich, USA) prepared in 1× PBS. Cells were washed twice after the incubation and resuspended in 500 µL 7H9. H_2_O_2_ added cells were used as a positive control. Fluorescence (Ex/Em: 450 nm/520 nm) was analyzed using a microplate reader (Tecan, Austria). Fluorescence was normalized to the OD.

### UV-Visible spectroscopy

UV-Visible spectral profiles were obtained to validate the interaction of PdtaS with hemin and Fe^2+^ using the microplate reader (Tecan, Austria). Spectral data were collected from 200–700 nm in 1 nm increments. Samples were run in a clear, flat-bottomed 96-well reaction plate using 10–20 µg of total protein and brought to a final reaction volume of 100 µL per well using 0.2 M Tris-HCl, pH 8.0. All spectral data were blank corrected using 100 µL of 0.2 M Tris-HCl, and 100 µL of hemin chloride (10 µM). All data were run in triplicate and the mean was used for subsequent analysis.

### Intracellular iron estimation

Intracellular iron was determined using the ferrozine assay. The protein samples were denatured using 0.2M HCl, followed by centrifugation at 12,000 rpm for 10′ to remove the precipitated protein. In the supernatant, 180 µL solution containing ascorbic acid (2.3 mM), ferrozine (0.3 mM), and 120 µL saturated ammonium acetate was added. The final solution was incubated for 30′ at room temperature. For measurement, the detection wavelength was 562 nm.

### Statistical analyses

The statistical analyses for significance were performed using ANOVA as mentioned in the figure legends. For all experiments, the number of independent biological replicates is indicated by “*n*.” The data for experiments are shown as mean ± SEM and analyzed using Graph Pad Prism 8.0 (GraphPad Software Inc, San Diego, CA, USA).

## Data Availability

Figures S1 to S8, Table S1 and Supplementary methods are available in Figshare (https://doi.org/10.6084/m9.figshare.26928703.v1).
